# Chronic Administration of 5-HT1A Receptor Agonist Relieves Depression and Depression-Induced Hypoalgesia

**DOI:** 10.1155/2014/405736

**Published:** 2014-01-23

**Authors:** Zhao-Cai Jiang, Wei-Jing Qi, Jin-Yan Wang, Fei Luo

**Affiliations:** ^1^Key Laboratory of Mental Health, Institute of Psychology, Chinese Academy of Sciences, Beijing 100101, China; ^2^University of Chinese Academy of Sciences, Beijing 100039, China; ^3^Department of Psychology, Guang'anmen Hospital, Chinese Academy of Chinese Medical Sciences, Beijing 100035, China

## Abstract

Previous studies have shown that depressed patients as well as animal models of depression exhibit decreased sensitivity to evoked pain stimuli, and serotonin is indicated to be involved in depression-induced hypoalgesia. The purpose of this study was to investigate the potential role of 5-HT1A receptor in the depression-induced hypoalgesia. Acute or chronic administration of 5-HT1A receptor agonist, 8-OH-DPAT, was performed in olfactory bulbectomy (OB) and sham-operated rats. The depression-like behavior and pain thresholds were measured using open-field test and radiant heat thermal pain test, respectively. We found that acute administration of 8-OH-DPAT increased locomotor activity and pain thresholds in the sham rats but had no effect on the OB rats. In contrast, chronic administration of 8-OH-DPAT reduced locomotor activity and pain thresholds and restored them to normal level. Increased pain thresholds were also observed in the sham rats after the chronic administration. These results demonstrated that chronic administration of 8-OH-DPAT reversed the depression-induced decrease in pain sensitivity in rats, suggesting that 5-HT1A receptor may play a role in the depression-associated hypoalgesia.

## 1. Introduction

Both depression and pain are debilitating diseases that lead to enormous demands on medical services and compromise the life qualities of patients. In clinical practice, studies have shown that a considerable proportion of patients with depressive disorders suffer from chronic pain [1] and vice versa, patients with chronic pain have an increased risk of developing depressive disorders [2]. In contrast to this close clinical association of pain and depression, depressive patients present decreased sensitivity to experimental pain stimulus [3, 4]. Animal research has also found that the pain thresholds are increased in rats with depressive-like behaviors [5–7].

Recent studies have indicated a potential role of serotonin in depression-induced hypoalgesia [8]. Serotoninergic neurotransmission has long been thought to be involved in the nociceptive processing as well as in the pathophysiology of depression [9]. In clinical practice, selective serotonin reuptake inhibitors (SSRIs) and serotonin-noradrenalin reuptake inhibitors (SNRIs) are commonly used to treat chronic pain [10]. Clinical studies have found that sleep deprivation therapy, which could increase serotoninergic transmission [11], reversed pain sensitivity towards hyperalgesia [8]. And six weeks of SNRIs duloxetine treatment reduced experimental heat pain thresholds to normal in patients with depression [3]. In our previous studies, it has been found that the thermal nociceptive thresholds in depressive-like rats approached normal level following systemic administration of SSRIs fluoxetine [5, 7]. However, no study so far has investigated further mechanism mediating these effects.

5-HT1A receptor is the most abundant serotonin subtypes expressed in the brain. It is widely distributed in regions such as prefrontal cortex, limbic system, and hypothalamus that receive serotonergic input from the raphe nuclei. It has been accepted that 5-HT1A receptor play a predominant role in the modulation of pain [12, 13]. Human studies suggested that chronic pain was associated with low 5-HT1A receptor [14]. In addition, lower 5-HT1A receptor densities were found in depressed rats [15] as well as depressed patients [16].

The present study was designed to investigate the potential role of 5-HT1A receptor in depression-induced hypoalgesia by administrating 5-HT1A receptor agonist 8-OH-DPAT in rats. The olfactory bulbectomized (OB) rat was used as the animal model of depression. We hypothesized that chronic administration of 8-OH-DPAT could relieve depressive-like behaviors as well as depression-induced hypoalgesia in the OB rats.

## 2. Materials and Methods

### 2.1. Animals

Eighty-one male Sprague Dawley rats (weight on arrival 200–220 g, Laboratory Animal Center of the Academy of Military Medical Sciences, Beijing, China) were used in this study and housed individually. Food and water were available *ad libitum*. The colony was maintained at 22 ± 2°C with a standard 12 h light-dark cycle (lights on at 07:00 am). Animals were allowed to habituate to the environment for 1 week before experiments and were handled daily by the experimenter. Adequate measures were taken to minimize pain or discomfort. Experiments were carried out in accordance with the National Institute of Health Guide for the Care and Use of Laboratory Animals (NIH Publications no. 80-23) revised 1996. The research protocol was approved by the Institutional Animal Care and Use Committee of Chinese Academy of Sciences.

### 2.2. Experimental Design

**Figure 1:**
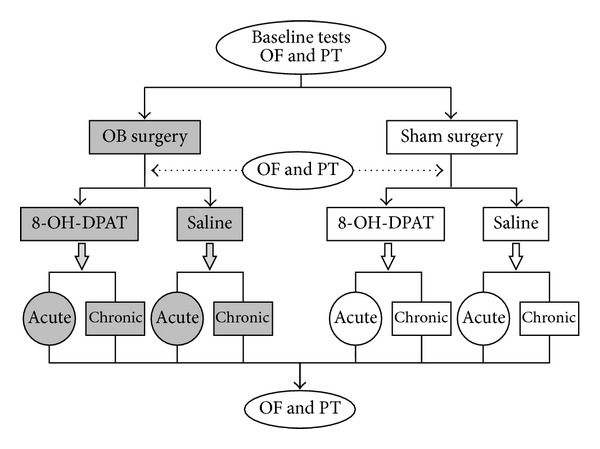
Schematic diagram of the experimental protocol. Baseline open-field (OF) activity and thermal pain thresholds were measured initially. Then rats were divided into two groups (OB and sham groups, receiving olfactory bulbectomy and sham surgery, resp.). Both groups were further divided into two subgroups for either saline or 8-OH-DPAT injection, which was administrated 30 min before behavioral tests (acute administration) or once daily for consecutive 14 days (chronic administration).

Two experiments were performed in this study. In experiment 1, 5-HT1A receptor agonist 8-OH-DPAT was intraperitoneally injected acutely 30 min before the open-field test and thermal pain threshold test. In experiment 2, 8-OH-DPAT was intraperitoneally administrated chronically for consecutive 14 days before behavior tests. Each rat was assigned to participate in only one experiment. The experimental protocol was depicted in Figure 1. For both experiments, rats were initially tested in the open-field and in the paw withdrawal latency (PWL) to noxious radiant heat. Then they were divided into two groups (OB group and sham group) and balanced according to their open-field locomotion and thermal pain thresholds. The rats in OB group and sham group underwent bilateral olfactory bulbectomy and sham surgery respectively. After 2-week recovery period, open-field test and pain threshold test were performed again to assess the depressive state and pain sensitivity of rats. Both groups were further divided into two subgroups: saline group (i.e., OB/saline and sham/saline) and 8-OH-DPAT group (i.e., OB/8-OH-DPAT and sham/OB/8-OH-DPAT), which received intraperitoneal injection of saline and 8-OH-DPAT respectively.

### 2.3. Surgical Procedure for Olfactory Bulbectomy

Animals were anesthetized with sodium pentobarbital (0.5 mg/kg, i.p.) and fixed on a stereotaxic apparatus (Stoelting, USA). A midline sagittal incision was made to expose the skull. Two 2-mm diameter holes were bored 8 mm rostral to the bregma and 2 mm lateral to the midline separately. The bilateral olfactory bulbs were sucked from the holes using a vacuum pump, and the cavity was filled with gel foam (Coltene Whaledent, Switzerland) to control bleeding. Special care was taken to avoid damaging the frontal cortex. Penicillin powder was sprinkled on the wound prior to closure. Sham-operated rats were treated similarly except that no brain tissues were removed. Body weight of each rat was measured the day before, and daily for 14 days after the surgery. At the end of the experiment, animals were dissected to check if all the olfactory bulbs were removed. If not, the data will be rejected in the final analysis.

### 2.4. Behavioral Tests

#### 2.4.1. Open-Field Test

The open-field test was performed in an iron circular black base (180 cm in diameter) and applied to analyze the locomotor behaviors of rats. The wall surrounding the base consisted of a 50 cm high iron sheet. Illumination was provided by a 40-W bulb. Each animal was tested in the open-field for 5 min. The distance traveled during the test was recorded by a computer-based system Etho Vision (Noldus Information Technology, Wageningen, the Netherlands). In the interval between each two tests, the apparatus was cleaned with ethanol and water to remove olfactory cues.

#### 2.4.2. Pain Threshold Test

The apparatus and test for thermal evoked pain were the same as described by Wang et al. [17]. In brief, rats were placed into a Plexiglas chamber on a glass floor, beneath which the radiant heat apparatus (100 W projector lamp) was situated. A beam of light through a hole (4 mm in diameter) of the apparatus was focused on the plantar surface of the left hindpaw. PWL was defined as the length of time between the light onset and the paw lift. The intensity of light was adjusted, so that the baseline PWL was around 7 s, with a cut-off time of 22 s to prevent tissue damage. A total of four trials were performed for each rat with at least 5-min interval. The last three trials were averaged to get a mean latency as the threshold of the thermal evoked pain.

### 2.5. Drugs

The 5-HT1A receptor agonist 8-OH-DPAT was purchased from Sigma-Aldrich (St. Louis, Missouri) and dissolved in saline (0.9% NaCl) immediately before application. Separate subgroups of bulbectomy and nonbulbectomy animals were treated with intraperitoneal injections of either 8-OH-DPAT (3 mg/kg) or saline (3 mg/kg). In experiment 1, one day after the depression model established, the open-field was tested with saline or 8-OH-DPAT injected 30 min before. Three days after the depression model established, paw withdrawal latency was tested with saline or 8-OH-DPAT injected 30 min before. In experiment 2, after the depression model established, consecutive 14-day injection of saline or 8-OH-DPAT once daily was performed according to groups. The open-field and radiant heat paw withdrawal latency were tested on the 15th day.

### 2.6. Statistical Analysis

GraphPad prism 5.0 was used to analyze data and generate graphs. Data involving 2 factors was analyzed with two-way analysis of variance (ANOVA) followed by Bonferroni post hoc test. Student's *t*-test was used for comparing means of two groups. The data was presented as means ± SEM. The statistical significance was set at *P* < 0.05.

## 3. Results

### 3.1. Behavioral Outcomes of the OB Depression Model

**Figure 2:**
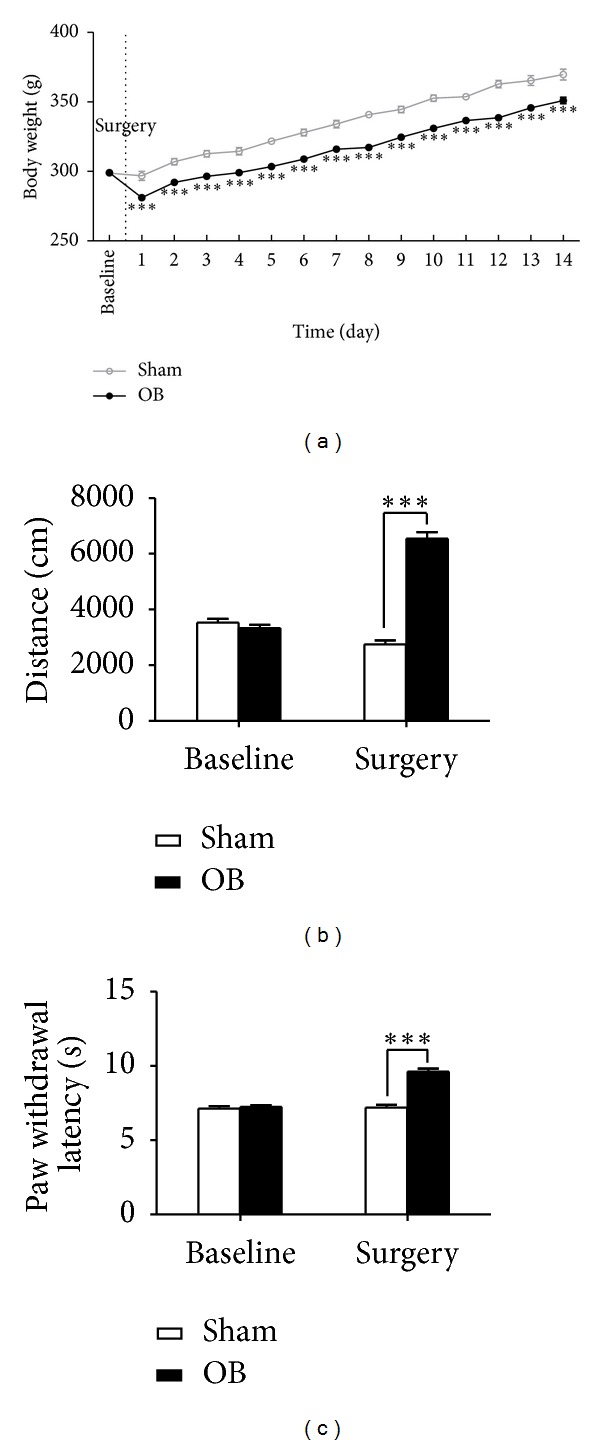
Behavioral outcome of the OB model for depression and depression-induced hypoalgesia. (a) Body weights. Significant decrease was observed in body weights in the OB group in comparison to the control group over the 14-day postoperation period (*n* = 33–48). (b) Open-field test. Significant higher level of locomotor activity was found in the OB rats than in the control rats (*n* = 33–48). (c) Thermal pain threshold test. The paw withdrawal latency to noxious radiant heat stimuli was significantly prolonged in the OB rats (*n* = 33–48). Data are presented as mean ± SEM.***P < 0.001.

As shown in Figure 2(a), the baseline body weights of animals did not differ between OB and sham groups. During the following two weeks of observation, significant reduction of weight gain was observed in the OB rats as compared to the control rats (two-way ANOVA, group effect: *F*(1, 1106) = 53.08, *P* < 0.001; Bonferroni posttests, *P* < 0.001 every day) (Figure 2(a)). After surgery, the OB rats showed significantly higher level of locomotor behaviors in open-field than that of control rats (two-way ANOVA, 6541 ± 238 versus 2746 ± 147 cm, *P* < 0.001) (Figure 2(b)). These results indicate that the OB rats have exhibited depressive-like behaviors and the animal model for depression has been successfully established.

In addition, animals in the OB group displayed longer PWLs compared to the control group (two-way ANOVA, 9.62 ± 0.20 versus 7.21 ± 0.19 s, *P* < 0.001) (Figure 2(c)), suggesting that the depressive-like rats had higher pain thresholds than the normal rats. That is, the OB-treated rats developed hypoalgesia to noxious thermal stimuli.

### 3.2. Effects of Acute Administration of 5-HT1A Receptor Agonist

**Figure 3:**
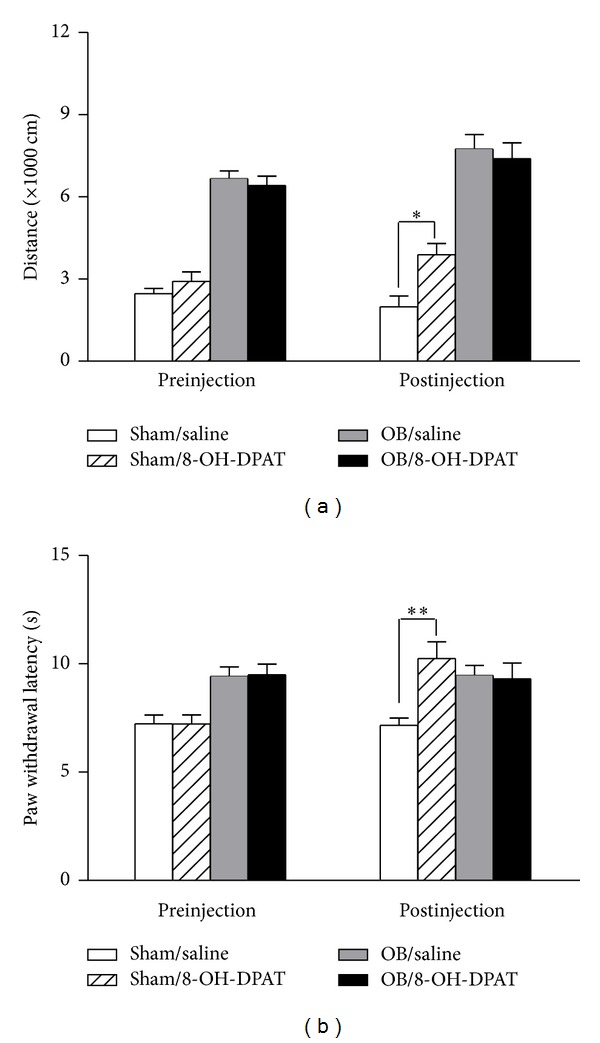
Effects of acute administration of 8-OH-DPAT. Acute 8-OH-DPAT treatment significantly increased locomotor activity (*n* = 8–12) (a) and pain thresholds (*n* = 8–12) (b) in the sham-operated rats but have no effect on the OB rats. Data are presented as mean±SEM.*P < 0.05, **P < 0.01.

As illustrated in Figure 3, acute 8-OH-DPAT treatment significantly increased the distance traveled in open-field in the control group (two-way ANOVA, 3889 ± 403 versus 1989 ± 396 cm, *P* < 0.05) but had no effect on the OB rats (Figure 3(a)). Additionally, the sham-operated rats exhibited significantly longer PWLs after acute 8-OH-DPAT treatment (two-way ANOVA, 10.24 ± 0.77 versus 7.15 ± 0.34 s, *P* < 0.01) and the OB rats did not show significant difference (Figure 3(b)). These results suggest that acute administration of 8-OH-DPAT increase locomotor activity and *per se* have antinociceptive effect on the normal rats but do not exert an influence on the depressive-like rats.

### 3.3. Effects of Chronic Administration of 5-HT1A Receptor Agonist

**Figure 4:**
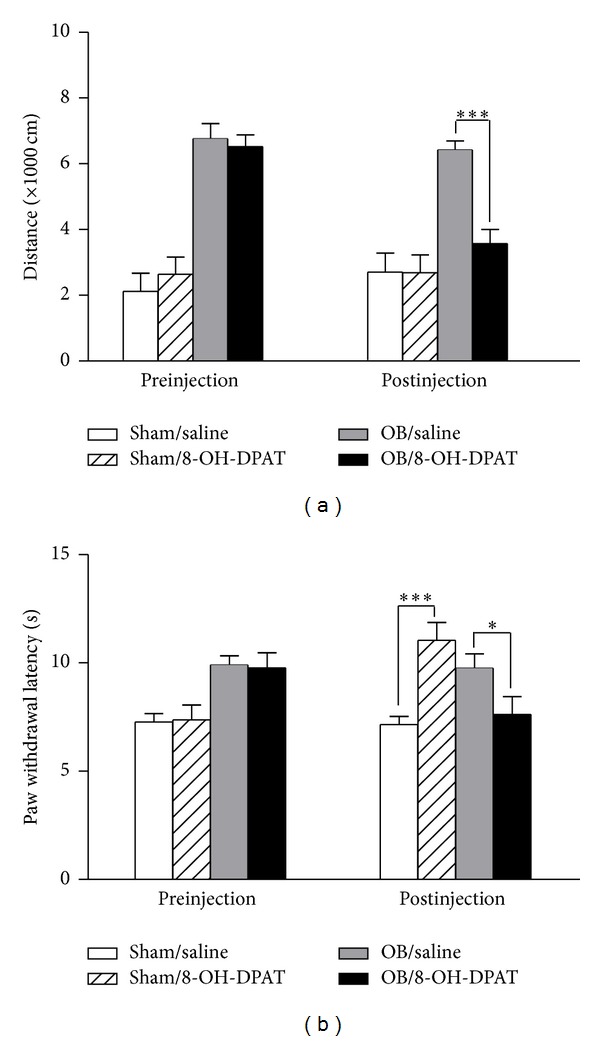
Effects of chronic administration of 8-OH-DPAT. (a) Locomotor activity in the open-field. Chronic 8-OH-DPAT treatment had no effect on the sham rats but significantly reduced the locomotor activity in the OB rats (*n* = 8–12). (b) Radiant heat pain threshold. Pain thresholds in the sham rats were increased after the treatment; by contrast, those in the OB rats were decreased and restored to normal level (*n* = 8–12). Data are presented as mean ± SEM. *P < 0.05, ***P < 0.001.

As shown in Figure 4, chronic 8-OH-DPAT treatment had no effect on the sham-treated rats, while significantly reducing the locomotor activity in the OB rats (two-way ANOVA, 3568 ± 438 versus 6425 ± 275 cm, *P* < 0.001), contrary to the observation of acute administration (Figure 4(a)). Thermal pain threshold test revealed significantly prolonged PWLs in the sham-operated rats after chronic 8-OH-DPAT treatment (two-way ANOVA, 11.04  ±  0.83 versus 7.16 ± 0.37 s, *P* < 0.001), consistent with that observed in the acute administration. However, after chronic 8-OH-DPAT treatment, the PWLs in the OB rats were significantly reduced (two-way ANOVA, 9.76 ± 0.66 s versus 7.62 ± 0.83 s, *P* < 0.05) and restored to normal level (OB/8-OH-DPAT versus Sham/saline: two-way ANOVA, 7.62 ± 0.83 versus 7.16 ± 0.37 s, *P* > 0.05) (Figure 4(b)). These results suggest that chronic 8-OH-DPAT treatment relieves the depression-like behavior and OB-induced hypoalgesia in rats.

## 4. Discussion

In the present study, we investigated the effects of acute and chronic administration of 5-HT1A receptor agonist, 8-OH-DPAT, on the open-field behavior and thermal evoked pain thresholds in the sham- and OB-treated rats. The results showed that (1) acute administration of 8-OH-DPAT increased the locomotor activity and pain thresholds in the sham rats but had no effect on the OB rats; (2) by contrast, chronic administration of 8-OH-DPAT reduced locomotor activity and pain thresholds in the OB rats and restored them to the normal level. Increased pain thresholds were also observed in the sham rats after the chronic administration. These findings demonstrated that 5-HT1A receptor was involved in mediating the depressive-like behavior as well as the associated hypoalgesia in rats.

Serotonergic dysfunction has been implicated in the underlying pathophysiology of depression and chronic pain [9]. Previous studies have indicated that acute and chronic administration of 5-HT1A receptor agonist seems to have different antinociceptive and antidepressive effects [13, 18]. In the present study, we tested the effects of acute or chronic administration, respectively. Obviously, in OB rats, only chronic administration of 8-OH-DPAT demonstrated antidepressive effect, represented as decreased activity in the open-field test and reduced pain threshold to the noxious thermal stimulation. We did not find an antidepressive effect of acute injection of 8-OH-DPAT on the OB rats. These results are consistent with the previous studies [19] that OB model is sensitive almost exclusively to chronic, but not acute, antidepressant treatment. There is also evidence that the 5-HT1A receptor densities have been greatly downregulated in the OB rats as compared to the sham-operated rats [15]. Thus, it is most likely that a single dose of 8-OH-DPAT is not sufficient to produce a therapeutic effect on the depressive-like rats.

In the present study, both acute and chronic administration of 8-OH-DPAT had antinociceptive effects on the sham rats, consistent with previous studies that systemic administration of 8-OH-DPAT produced analgesia in the hot plate test and tail flick thermal pain test [20, 21]. A recent study in knockout mice has demonstrated that 5-HT1A receptors mediate an endogenous inhibitory control of heat-evoked nociception [22]. 5-HT1A receptors are located both presynaptically in the raphe nuclei and postsynaptically in discrete brain regions, including the cortex, amydale, and the hippocampus [16]. Evidence has supported that both pre- and post-synaptic 5-HT1A receptors are involved in the 8-OH-DPAT's analgesic action [23].

In addition, we observed reduced hypoalgesia to the normal level after chronic administration of 8-OH-DPAT in OB rats. Serotonergic drugs are commonly used as antidepressants in clinical practice. Antidepressants have been shown to improve depression and pain symptoms somewhat independently in the depressed patients. For example, approximately 50% of the improvement in pain intensity produced by duloxetine was independent of the improvement in depression [24]. In our study, the symptoms of depression and hypoalgesia seemed to be uniformly influenced by olfactory bulbectomy; thus, the reduced hypoalgesia may be attributed to the indirect result of 8-OH-DPAT-induced antidepressive effect. Deficiency in serotonergic function has been demonstrated to be the primary pathogen of depressive disease [9]. It has been reported that chronic administration of 5-HT1A agonist induced desensitization of presynaptic 5-HT1A receptors in the raphe nuclei and thereby enhanced the 5-HT release [25]. Moreover, an overactivity of hypothalamo-pituitary-adrenal (HPA) axis has been found in depressive patients [26]. Activation of the 5-HT1A receptors has been demonstrated to normalize the HPA activity thus to exert a therapeutic effect [27].

Another possible mechanism underlying the restored pain sensitivity in OB rats after 8-OH-DPAT treatment may be due to the inhibition of opioid activity. Stress-evoked release of opioid has been demonstrated to inhibit pain in depressed patients [28]. Previous studies have shown that 5-HT1A receptor agonist can decrease opioid-mediated analgesia and tolerance [13]. It has also been found that 8-OH-DPAT could inhibit endogenous opioids release evoked by electrical stimulation in rat spinal cord slices [29]. Thereby, chronic administration of 8-OH-DPAT might restore depression-induced hypoalgesia by reducing the endogenous opioids release.

In sham rats, we found that acute administration 8-OH-DPAT increased the locomotor activity. The increase in the distance accorded with other reports that acute administration of 5-HT1A agonists caused serotonin syndrome represented by hyperactive behavior, flat body posture, and forepaw treading [30, 31]. It has been reported that low dose of 8-OH-DPAT (0.01–0.05 mg/kg) preferentially stimulates 5-HT1A autoreceptors, while high dose (i.e, ≥0.2 mg/kg) could activate postsynaptic receptors [30, 31], which increase 5-HT release and produced hyperactivity [30]. Thus, the relatively high dose used in this study may account for the hyperactivity of rats. As previous studies have shown that repeated doses of 8-OH-DPAT could reduce the serotonin syndrome through desensitization of postsynaptic 5-HT1A receptors [23, 32], our study confirmed the prior findings by showing that chronic administration of 8-OH-DPAT did not lead to hyperactive behavior in the open-field in sham rats.

In summary, our results demonstrated that 5-HT1A receptor was involved in the OB-induced depressive-like behavior and hypoalgesia in rats. Nonetheless, our study has several limitations. (1) Only one relatively high dose of 8-OH-DPAT (3 mg/kg) was adopted in this study. As mentioned above, different doses of 8-OH-DPAT may exert differential effects. Thus, more experiments involving various doses need to be done in future studies. (2) Only radiant-heat evoked pain model was used in this study. Given that depression can cause alleviated evoked pain and aggravated spontaneous pain [5–7], more pain models should be included to reveal the role of 5-HT1A receptor in the depression-related behaviors.

## 5. Conclusion

In conclusion, our study demonstrated that chronic administration of 5-HT1A receptor agonist 8-OH-DPAT relieved depression and depression-induced hypoalgesia, which suggested that 5-HT1A receptor may play key role in depression-induced hypoalgesia. Future studies should be designed to investigate more exact mechanism to build better strategies for the treatment of depression and chronic pain.
